# Biological Studies on the Fungi of the Human Mouth*German mycologists use the term ‘Pilz’ indiscriminately to designate either Schizomycetes, Blastomycetes, Hyphomycetes or Myxomycetes. When it is desirable to refer to any one of these groups in particular, they use the prefixes Spalt, Spross, Schimmel or Faden, and Schleim, giving Spaltpilz, Sprosspilz, Schimmel–or Fadenpilz and Schleimpilz. Following their example, I have in previous papers used the term fungus for all of the four groups of mycetes mentioned above, and shall also use the term in this paper, in which only Schizomycetes are treated of.

**Published:** 1885-05

**Authors:** W. D. Miller

**Affiliations:** Berlin


### Independent Practitioner.

VOL. VI. MAY, 1885. No. 5.

### Original Communications.

### BIOLOGICAL STUDIES ON THE FUNGI OF THE HUMAN MOUTH.*

BY PROF. DR. W. D. MILLER, BERLIN.

In order to be able to determine upon the proper course to be taken in the attempt to remove or check the progress of any disease, it is necessary that our ideas of the cause and course of that affection be established upon the most certain, exact and scientific data which we are capable of attaining. Unfortunately for the dental profession, the attempt to furnish a scientific solution of the problems of dental caries has, until recently, been confined to a very few, and even now a majority of the investigators in dental pathology are content to restrict their observations to the clinical aspect of the question, a course which could never produce a satisfactory solution; while others even openly advocate a speculative course, and do not hesitate to ascribe to every new factor discovered in nature, a role in the production of caries of the teeth.

Consequently we have had presented to us, in turn, worms, acids, inflammation, electricity, infusoria, bacteria, putrefaction, toxic

*German mycologists use the term 'Pilz' indiscriminately to designate either Schizomycetes, Blastomycetes, Hyphomycetes or Myxomycetes. When it is desirable to refer to any one of these groups in particular, they use the prefixes Spalt, Spross, Schimmel or Faden, and Schleim, giving Spaltpilz, Sprosspilz, Schimmel-or Fadenpilz and Schleimpilz. Following their example, I have in previous papers used the term fungus for all of the four groups of mycetes mentioned above, and shall also use the term in this paper, in which only Schizomycetes are treated of.

agents, etc., etc., as causes or conditions of *caries dentium*, some of these theories containing some truth and some a surprising amount of absurdity. In the last two or three years, however, a great advance has been made in the methods of study, and a number of important points have been firmly established.

1. The observation of Leber and Rottenstein that micro-organisms are constantly present in decaying dentine, has been confirmed. (Weil, Milles, Underwood, Miller.)
2. The softening of dentine in caries has been shown to be chemically identical with that produced by certain weak organic acids. (Miller, Jeserich, Bennefeld.)
3. It has been established that various organisms found in the human mouth, produce the decalcifying acid by first converting non-fermentable sugars into fermentable varieties, and secondly, by splitting fermentable sugars into lactic acid. (Miller, Hueppe.)
4. The same organisms have been found capable of dissolving decalcified dentine, while they have no apparent effect, even after two or three years, on sound dentine. (Miller.)
5. Caries of dentine, chemically and morphologically identical with natural caries, has been produced outside of the mouth. (Miller.)
6. It has been furthermore shown that certain of the organisms of the human mouth are capable of developing under exclusion of air, thus making it possible for them to propagate within the substance of the dentine. (Miller, Hueppe.)

I propose to describe, in this and the following article, a series of experiments made for the purpose of obtaining more definite information respecting the number and morphology of the fungi of the human mouth, and their physiology, as far as is necessary to an understanding of the part which they may perform in the production of caries of the human teeth.

At the meeting of the American Dental Association, at Saratoga, a number of tubes containing pure cultures of fungi were passed around; with regard to these a reporter remarked that "they were evidently beyond the information of the majority." It is not very flattering to American dentistry if its representative Association allows a question of so great importance to remain beyond its comprehension, nor is there any excuse for such a condition of things now, so widespread have the methods of pure culture become. I

rather incline to the opinion that the reporter misinterpreted the apathy of the members of the Society. I shall, at any rate, here describe in a few words the methods now universally employed in isolating any given fungus, and then, more in detail, give the means which I have used to ascertain the physiological characteristics of the different fungi when obtained in pure culture.

We will start with a solution densely impregnated with micro-organisms, and a number of tubes of culture gelatine, perfectly sterilized. The gelatine being melted, we add to the first tube one bead (on a loop of sterilized platinum wire) of the solution. This is called the *first dilution.* From this tube we add two or three beads to a second tube (*second dilution*), and from the second five or six beads to a third tube (*third dilution.*) The gelatine is then poured upon horizontally placed, sterilized, cold glass plates. It congeals in a few seconds, and the three plates are placed in a pile (on glass benches) in a moist cell. The plates are examined after twenty-four to thirty-six hours, under a magnification of one hundred diameters.

By this means the fungi are so separated that on the third plate there will generally not be more than two to ten; (on the second there may be one hundred or two hundred, while on the first, of course, there are very many more.) As each micro-organism develops, being fixed in the gelatine, we will have at that point a pure culture of that particular kind. At another point we obtain a colony of a second kind, and so on. In general, colonies of different fungi may be distinguished with the greatest ease, by their microscopic appearance. With a sterilized platinum wire, bent at right angles at the end, we now pick up a number of the colonies of each kind, under the microscope (one hundred diameters), and transfer them directly to tubes of culture gelatine, only one colony to each tube. We have then (except in case of a possible accidental air-infection) pure cultures. Some experience is necessary to enable one to pick up the colonies under the microscope. Beginners should not attempt it with plates where more than one colony is in the field at once.

The method described in this journal (page 340, 1884), may also sometimes be used to great advantage. For fungi which do not grow on gelatine, Agar-Agar, or congealed blood serum, should be used. The former, one to one and a half per cent, has a higher melting point than gelatine, ten per cent, and remains solid at the temperature of the human blood. When it is used for plate-cultures, it must be melted in hot water, and the infection made at a temperature of 40° to 42° C. Below this temperature it becomes solid, and cannot be poured; above it the germs would be liable to suffer. In other respects the Agar-Agar media are treated as the gelatine. Congealed blood serum cannot, of course, be poured upon plates. It is prepared in test tubes so inclined as to give the greatest possible surface, and a minimum quantity of the substance containing the fungus or fungi spread over the surface. Having obtained a pure culture of any fungus, the points to be determined regarding it are the following:

1. Its morphology; (bacillus, spirillum, micrococcus.)
2. Is it moveable? does it produce spores?
3. What are its growth-characteristics on various media, microscopically and to the naked eye?
4. What are its relations to oxygen?
5. Does it produce fermentation? If so, what fermentation, under what conditions, and with or without development of gas?
6. Does it cause putrefaction?
7. Does it have a diastatic, inverting, or peptonizing action?
8. Has it a pathogenic character?
9. Does it produce coloring matter?
10. What is its susceptibility to the action of the various antiseptics?

The first and second of these questions are, of course, determined by the microscope alone; the third, by the microscope and the naked eye combined; the fourth by the methods described in this journal, page 62, 1884, or by placing a thin strip of mica upon one half of the culture-plate before the gelatine solidifies; the mica then adapts itself closely to the surface of the gelatine, excluding the air, and if the fungus requires oxygen for its development the colonies beneath the mica either will not develop at all, or they will be very small compared with those on the other half of the plate, their growth ceasing as soon as the oxygen in the gelatine has been consumed. (Koch.) The fifth point is answered by infecting fermentable solutions with the fungus in question, placing it under various conditions of temperature, etc., and determining the products of fermentation (if any); the sixth by analogous methods; the seventh question is determined by the action of the fungi upon starch, cane sugar, and albumen, (boiled white of egg); the eighth by experiments on animals; the ninth by the appearance or non-appearance of color in the vegetation itself, or in the surrounding medium; the tenth by experiments that will readily suggest themselves.

Other points to be investigated will be mentioned further on. Boiled potato is a medium of great value in the determination of Schizomycetes. No medium, however, requires greater care in preparation and after treatment than this, in order to obtain satisfactory results. Any sound potato *which does not become mealy or crack open on boiling,* will do for the purpose; it is first thoroughly washed and brushed, and all defective spots and deep eyes being removed, it is placed for one hour in a corrosive sublimate solution, five to one thousand, then in the steam sterilizer for one-half to one hour. In the mean time the moist cell is sterilized, and the bottom lined with filter paper wet with sublimate solution, five to one thousand. The potatoes are, while hot, removed from the sterilizer with sterilized forceps, cut into halves with a cold sterilized knife, and placed directly upon the sublimate paper (the cut surface up), and the cell closed. Potato sections prepared in this way should remain unchanged indefinitely. When the potato has become cool, the cover of the cell is carefully removed, and the fungus which is to be cultivated is spread upon a space about as large as a dime, in the centre of the section. Fungi which, morphologically as well as in their reaction upon gelatine, Agar-Agar and blood serum, show no appreciable differences, may sometimes be easily distinguished by aid of the potato culture. The potato can seldom be used to separate fungi, (*i. e.* to prepare pure culture). It is chiefly used as a reagent in distinguishing between fungi already in pure culture. For example, all comma bacilli yet discovered grow on potato, except the one found by Dencke in old cheese, which does not develop at all on potato, and is thereby at once distinguished as an entirely different fungus.

Eggs may often be used to great advantage. They are prepared as follows. The *fresh* egg is placed in sublimate, five to one thousand, for ten minutes, then in the steam sterilizer for one hour. The cell for eggs is prepared as for potatoes, except that a sterilized glass plate, resting on a glass bench, is placed in the bottom to support the egg sections. As the eggs must be handled with the fingers, the hands must be thoroughly washed, then soaked in sublimate, five to one thousand, and then washed again in *alcohol absolutus*, to remove the sublimate. The eggs are shelled while still hot, and cut into two, three, or four sections. They are vaccinated in points upon the white; the yellow is not so well adapted to culture experiments, since it cannot be cut with a smooth surface.

I always keep on hand sections of potato and egg, also tubes of gelatine, Agar-Agar, and blood serum, and when in my practice particularly good material, or anything uncommon presents itself, a portion of it is at once transferred to these different culture media, so that it is pretty sure to develop in one of them, at least. For example, I have several times met with a fungus in the human mouth which produces a yellowish coloring matter, and which absolutely refuses to grow on anything which I have tried, except potato.

(TO BE CONTINUED.)

